# Efficacy of insulin targeted gene therapy for type 1 diabetes mellitus: A systematic review and meta-analysis of rodent studies

**DOI:** 10.22038/ijbms.2020.39470.9359

**Published:** 2020-04

**Authors:** Moosa Rahimi Ghiasi, Hamed Mohammadi, Michael E. Symonds, Seyed Mohammad Bagher Tabei, Ahmad Reza Salehi, Sima Jafarpour, Leila Norouzi Barough, Elnaz Rahimi, Zohreh Amirkhani, Maryam Miraghajani, Rasoul Salehi

**Affiliations:** 1 Department of Genetics and Molecular Biology, School of Medicine, Isfahan University of Medical Sciences, Isfahan, Iran; 2 Food Security Research Center, School of Nutrition and Food Science, Isfahan University of Medical Sciences, Isfahan, Iran; 3 The Early Life Research Unit, Division of Child Health, Obstetrics and Gynaecology, and Nottingham Digestive Disease Centre and Biomedical Research Centre, School of Medicine, University of Nottingham, Nottingham, UK; 4 Department of Genetics & Maternal-Fetal Research Center, Shiraz University of Medical Sciences, Shiraz, Iran; 5 Cancer Research Center, Shahid Beheshti University of Medical Sciences, Tehran, Iran; 6 Pediatric Inherited Diseases Research Center, Isfahan University of Medical Sciences, Isfahan, Iran

**Keywords:** Gene therapy, Insulin, Meta-analysis, Non-viral vector, Type 1 diabetes mellitus, Viral vector

## Abstract

Diabetes mellitus (DM) is a major worldwide public health challenge, for which gene therapy offers a potential therapeutic approach. To date, no systematic review or meta-analysis has been published in this area, so we examined all relevant published studies on rodents to elucidate the overall effects of gene therapy on bodyweight, intraperitoneal glucose tolerance test (IPGTT), fasting blood glucose, and insulin in animals with type 1 DM. The Cochrane Library, PubMed, Embase, ISI Web of Science, SCOPUS, and Google Scholar were systematically searched for potentially relevant studies. Mean±standard deviation (SD) was pooled using a random-effects model. After the primary search, out of 528 studies identified, 16 studies were in concordance with predefined criteria and selected for the final assessment. Of these, 12 studies used viral manipulation, and 4 employed non-viral vectors for gene delivery. The meta-analysis showed gene therapy with a viral vector decreased mean IPGTT (-12.69 mmol/l, *P<*0.001), fasting blood glucose (-13.51 mmol/l, *P<*0.001), insulin (398.28 pmol/l, *P<*0.001), and bodyweight (24.22 g, *P<*0.001), whereas non-viral vectors reduced fasting glucose (-29.95 mmol/l, *P<*0.001) and elevated insulin (114.92 pmol/l, *P<*0.001). Gene therapy has favorable effects on alleviating type 1 DM related factors in diabetic rodents.

## Introduction

Diabetes mellitus (DM) is one of the most important public health challenges worldwide (1, 2), of which type 1 diabetes mellitus (T1DM) (3, 4) is characterized by hyperglycemia caused by autoimmune destruction of pancreatic β-cells, the main site of insulin production and secretion (4). The prevalence of diabetes was estimated to be 422 million in 2014 (1) and expected to reach 522 million in 2030 (5). The total cost of diabetes and pre-diabetes in the US is $322 billion per annum (6), and health care costs for people with diabetes are 2.3 times higher than those without diabetes (7). Most of the global burden of this disorder is due to morbidity and mortality that arises from complications of the disease (1, 8-13).

The most commonly utilized treatment for T1DM is insulin infusion, which requires close monitoring of blood glucose during insulin therapy, which is then needed daily and reduces patient compliance (14). At the same time, the optimal blood glucose is rarely achieved and patients remain at risk from experiencing regular periods of hypo or hyperglycemia (15, 16). This type of adverse blood glucose places the patient at risk of hypoglycemic coma or hyperglycemia complications, such as retinopathy, nephropathy, neuropathy, and cardiovascular disease (16, 17). Consequently, a treatment option that is more able to maintain normoglycaemia without adverse complications and greater compliance remains highly desirable (18, 19). Insulin gene therapy is one alternative and represents a novel therapeutic approach to achieving regulated insulin production and delivery (20). Recently, numerous studies have reported the effects of insulin gene therapy on T1DM, which showed some beneficial outcomes in some (21, 22), but not all cases (23). The aim of this paper was to conduct a systematic review and meta-analysis to estimate the effects of insulin gene therapy on T1DM related factors, including bodyweight, intraperitoneal glucose tolerance test (IPGTT), fasting blood glucose, and insulin in diabetic rodents.

## Materials and Methods

***Search strategy***

A comprehensive search was conducted in medical databases including Cochrane reviews, Medline/PubMed, EMBASE, ISI Web of Science, SCOPUS, and Google Scholar up to July 2019 using the following medical subject headings (MeSH) and non-MESH keywords relevant to ((“ Gene Therapies “[tiab] OR “ DNA Therapy “[tiab] OR “ Genetic Therapies “[tiab])) AND ((“Genetic Vectors”)) AND ((“diabetes mellitus”[tiab])) AND ((“insulin”[tiab])) regardless of language. The reference lists of related articles were then hand-searched for additional relevant studies. Titles/abstracts were screened for relevant studies by two independent investigators.

***Study selection***

*Inclusion and exclusion criteria*

Studies were eligible for inclusion in the current analysis if: (i) insulin gene therapy was assessed in animal studies; (ii) their final outcome was diabetes-related factors; and (iii) mean, standard error (SE), or standard deviations (SD) for the mentioned factors were provided. Studies were excluded if they did not include outcome measurements for diabetic control groups or only reported the mean average outcome during the treatment. Two investigators extracted data independently, and any discrepancies were resolved by discussion.

***Outcomes***

Studies evaluating insulin gene therapy effect on diabetes-related factors were included in the current study, and the outcomes of interest were IPGTT, fasting blood glucose, insulin, and bodyweight. 

***Data extraction***

The data included the first author, year of publication, country where the study was conducted, sample size, gene delivery route, gene delivery method, target tissue, follow-up duration, main outcome, covariates adjusted for in the analysis and mean and SD or SE. Characteristics of each study on insulin gene therapy by viral and non-viral vector are summarized in Tables 1 and 2, respectively.

***Statistical methods***

Means after treatment and their SD or SE were collated as the measurable effect of insulin gene therapy on diabetes-related factors. Meta-analysis was performed using the random-effects model and presented as forest plots. Evidence for publication bias was sought by performing Egger’s test in addition to visual inspection of the funnel plots. The percentage of variability across the pooled estimates attributable to heterogeneity beyond chance was estimated with the *I*^2 ^index, and the *P*-value for heterogeneity (I^2 ^>50% was considered as significant heterogeneity). In the case of significant between-study heterogeneity, sensitivity analyses were performed excluding individual studies to obtain an understanding of the reasons for any differences. Also, where there was a high likelihood of differences beyond chance, subgroup analysis, based on the gene delivery method and follow-up duration, was performed. 

Publication bias was assessed statistically by Begg’s test. *P*<0.05 was considered statistically significant. Statistical analyses were conducted using the statistical software package Stata (ver. 11.2). 

## Results

***Search results and characteristics of included studies***

**Figure 1:**
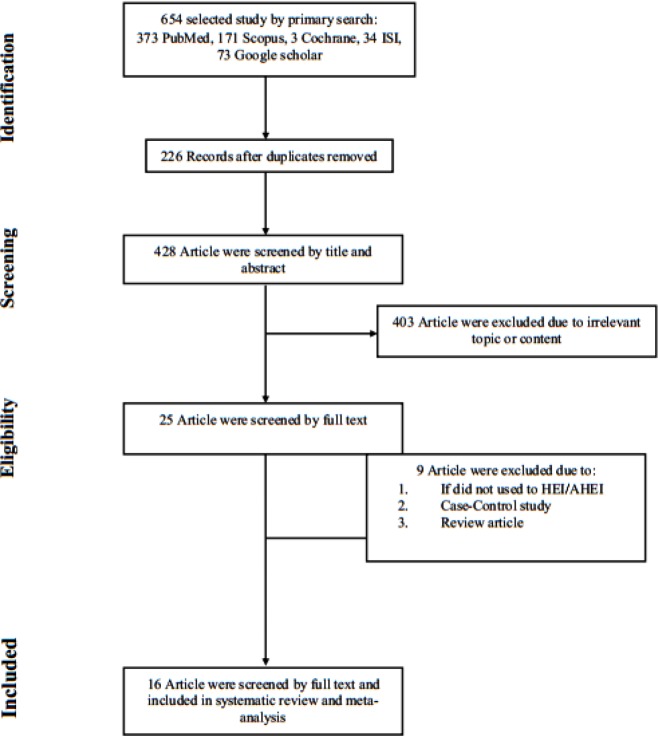
The flow diagram of study selection

The literature search on the subject of gene therapy and diabetes-related factors yielded 654 articles, of which 33 were reviewed as full texts. Of these, 16 studies met the inclusion criteria. The flow diagram summarizes the results of the study selection process for this meta-analysis (Figure 1). Out of 16 studies published 5 (23,27-28, 32, 35) were conducted in the USA, and 2 (22, 23), 2 (21, 29), 2 (31, 34), 2 (25, 26), 1 (36), 1 (30), 1(24) studies were published respectively in Germany, Australia, Korea, China, Italy, Taiwan, and Malaysia. Six (21, 22, 28-31), 13 (21-29, 31-34), 5 (21, 28, 29, 32, 34), and 11 (21, 23-26, 29-31, 34-36) studies assessed the effects of insulin gene therapy on IPGGT, FBS, bodyweight, and blood insulin, respectively. The sample size ranged from 3 animals to 18 with follow-up ranging from 2 min to 3 months. Target tissue was based on the liver in all of the viral vector studies and K-cell in non-viral vector studies. The gene delivery method was a viral vector in 11 studies (21, 22, 28, 32, 33, 36), which used the portal vein as the route of delivery. Others (29-31, 34, 35) used the hepatic artery and tail vein. Four studies used a non-viral vector as a gene delivery method, of which 2 (25, 26) used coloclysis as the route of delivery. The remaining used an oral route (24) or the pronuclei of fertilized mouse embryos (27). 

***Findings from the systematic review:***

Some studies that were initially included were subsequently excluded and were reported in a systematic study. 

Hsu *et al.* reported effect of insulin gene therapy on IPGTT; the IPGTT difference before glucose administration and 150 min after glucose administration in treat group was 1.21 mmol/l but in control diabetic group it was 10.9 mmol/l throughout the experimental period. Therefore, glucose was significantly decreased in the treat group. Similarly, the insulin level in the treat group was significantly increased (30.6±2.1 pmol/l) compared to the control group (30).

Another study showed that insulin gene therapy affected IPGTT and that the difference in blood glucose before and after insulin gene therapy in the treat group was 9.54±1.2 mmol/l (15.04±1.6 mmol/l to 5.49±0.4 mmol/l), whereas in the normal control group it was 5.55±0.6 mmol/l throughout the experimental period. Therefore, glucose was significantly decreased without any apparent significant differences in insulin (23). However, Rasouli *et al.* reported insulin gene therapy by GIP/Ins/pBud increased insulin in comparison to controls (1.048 pmol/l) (24). Similarly, Cheung used GIP/Ins fragments and injected them into pro-nuclei of fertilized mouse embryos. In the transgenic mice mean blood glucose and human insulin was 9.52±1.16 mmol/l and 39±16.9 pmol/l, respectively, which showed decreased blood glucose and raised human insulin (27). 

***Findings from the meta-analysis on insulin gene therapy and IPGGT***

*Gene therapy by viral vector*

**Figure 2:**
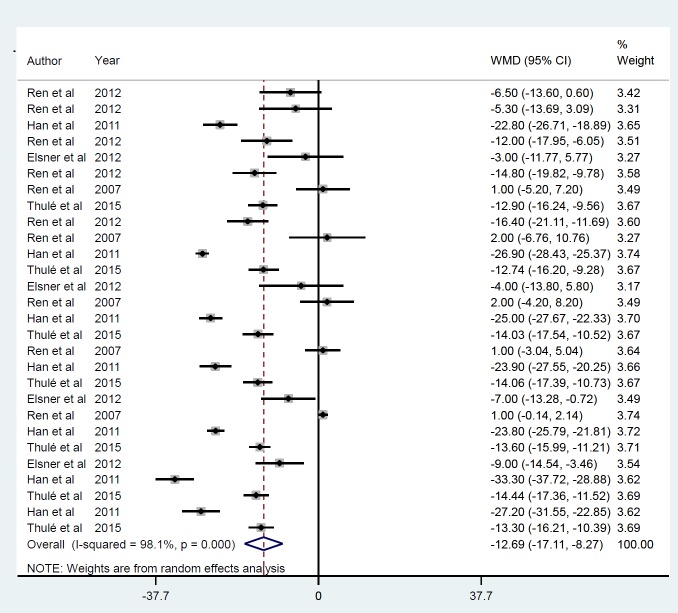
Mean of IPGGT after insulin gene therapy by viral vectors

**Figure 3:**
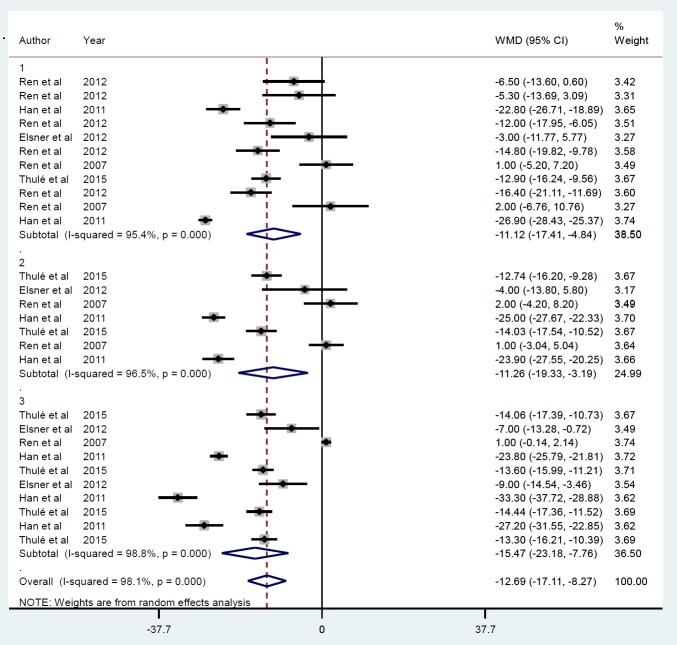
Subgroup analysis by follow-up duration for the mean of IPGGT after insulin gene therapy by viral vectors

**Figure 4:**
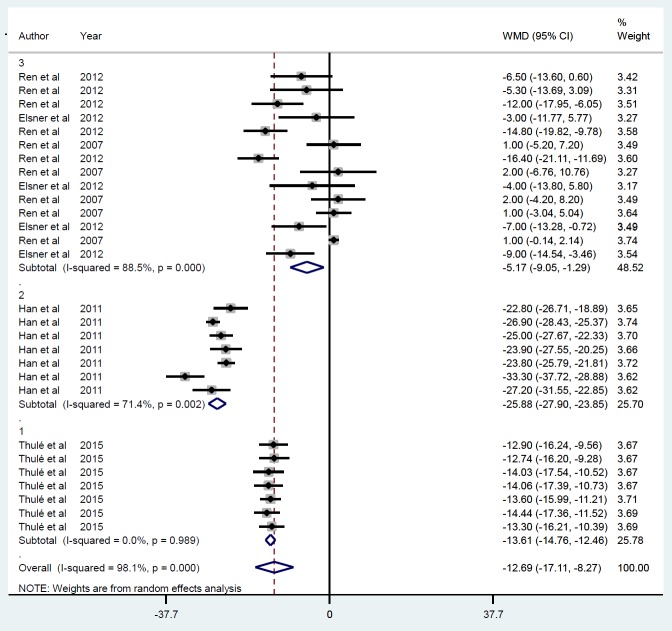
Subgroup analysis by gene delivery methods for the mean of IPGGT after insulin gene therapy by viral vectors

Five studies were identified (21, 22, 28-30), including 28 datasets that met the inclusion criteria based on their mean IPGGT after insulin gene therapy by viral vector that was reduced on average by -12.69 mmol/l (*P*<0.001) (Figure 2). Publication bias was observed (*P*=0.007) after using the trim-and-fill method to adjust for funnel plot asymmetry, although these results were unchanged. Between-study heterogeneity was also found (*I*^2^=98.1, *P*<0.001). The sensitivity analysis revealed that the exclusion of any single study did not alter the overall effect. For each study, assessment follow-up duration after treatment was classified as ≤ 30 min (ID=1), ≥ 60 and ≤ 90 min (ID=2), and ≥ 120 and ≤ 300 min (ID=3). Such subgroup analysis (Figure 3) showed no heterogeneity between studies, although the summary mean for IPGGT after ≥ 120 and ≤ 300 min was lower (-15.46 mmol/l, *P*<0.001) than achieved at ≤ 30 min (-11.12 mmol/l, *P*=0.001) and ≥ 60 and ≤ 90 (-11.25 mmol/l, *P*=0.006). The gene delivery method (AAV) (ID=1), r Adeno (ID=2) and lentiviral (ID=3), modified the IPGGT response that was greatest with the r Adeno method, (-25.87 mmol/l, *P*<0.001) compared to AAV (-13.60 mmol/l, *P*<0.001) and the lentivirus (-5.17 mmol/l, *P*<0.009) (Figure 4).

***Findings from the meta-analysis on insulin gene therapy and FBS:***

*Gene therapy by viral vector*

**Figure 5:**
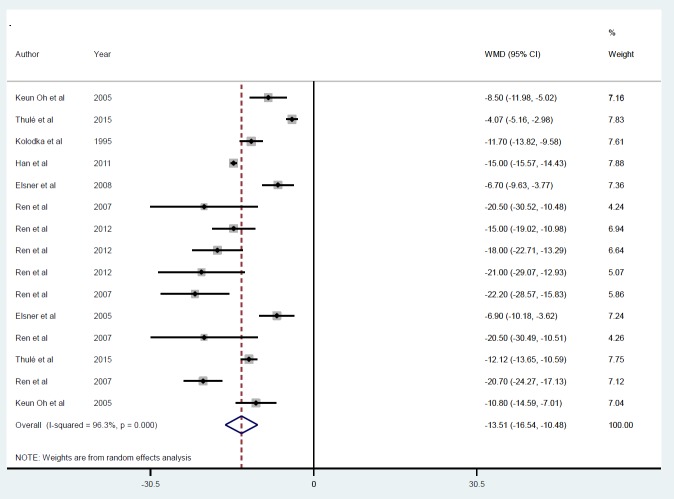
Mean of FBS after insulin gene therapy by viral vectors

**Figure 6:**
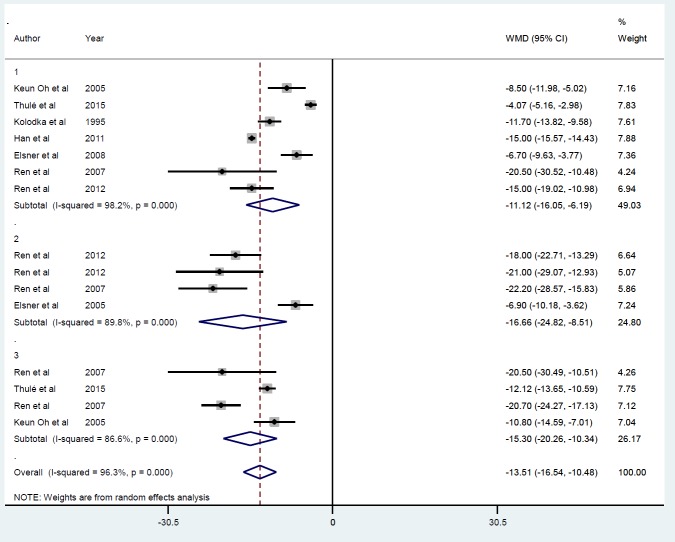
Subgroup analysis by follow-up duration for the mean of FBS after insulin gene therapy by viral vectors

Eight studies with 15 datasets describing the effects of insulin gene therapy by viral vectors gave a mean reduction in FBS (-13.51 mmol/l) (21, 22, 28, 29, 31-34) (Figure 5). No evidence of publication bias was found (*P*=0.86). The *I*^2 ^value indicates 96.3% of the variability was accounted for across the pooled estimates. Sensitivity analysis showed the exclusion of each study from the analysis did not change the overall effect. Subgroup analysis by follow-up duration confirmed a prominent effect (*P*<0.001) of insulin gene therapy on FBS at ≤ 5 days (ID=1), ≥ 10 and ≤ 30 days (ID=2), and ≥ 50 and ≤ 70 days (ID=3) (Figure 6). Insufficient studies in AAV, retroviral, and viral subgroups meant analysis according to gene delivery method was not possible. 

*Gene therapy by non-viral vector*

**Figure 7:**
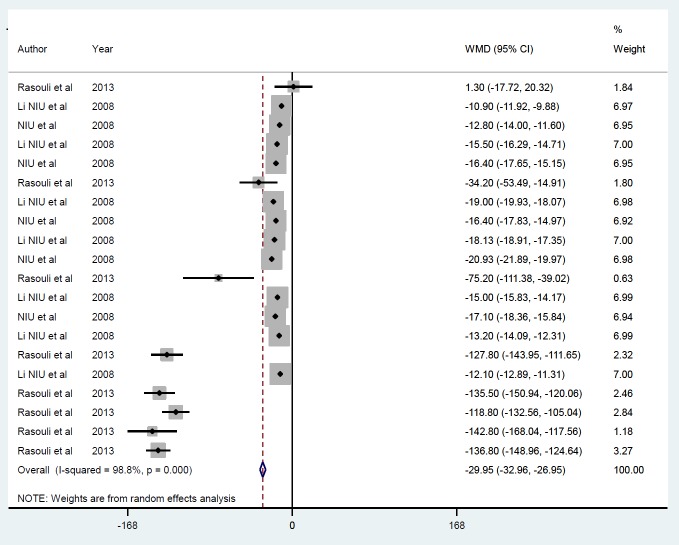
Mean of FBS after insulin gene therapy by non-viral vectors

**Figure 8:**
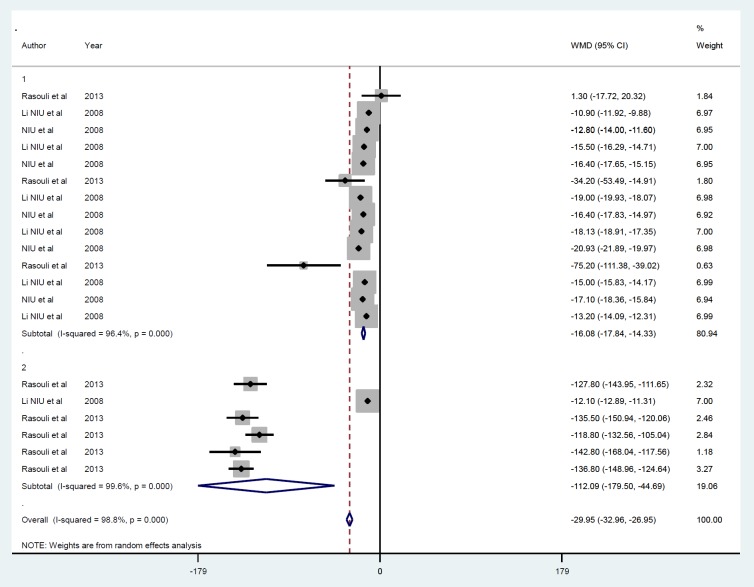
Subgroup analysis by follow-up duration for the mean of FBS after insulin gene therapy by non-viral vectors

Meta-analysis from three studies including 20 datasets that used non-viral vectors (24-26) also showed reduced FBS (-29.95 mmol/l, *P*<0.001) (Figure 7). Egger’s test was significant (*P*=0.001), but applying trim and fill had no effect on the outcome, as there was between-study heterogeneity (I^2 ^=98.8, % P=*P*<0.001). The exclusion of each study from the meta-analysis did not impact the overall sensitivity analysis. According to follow-up duration, studies were categorized into ≤ 1 day (ID=1), ≥ 7, and ≤ 15 days (ID=2) (Figure 8), and the magnitude of effect increase with time of follow up, i.e., FBS at ≤ 1 days, -16.08 g (*P*<0.001) compared with ≥ 7 and ≤ 15 days, -112.09 g (*P*<0.001).

***Findings from the meta-analysis on insulin gene therapy and blood insulin***

*Gene therapy by viral vector*

**Figure 9:**
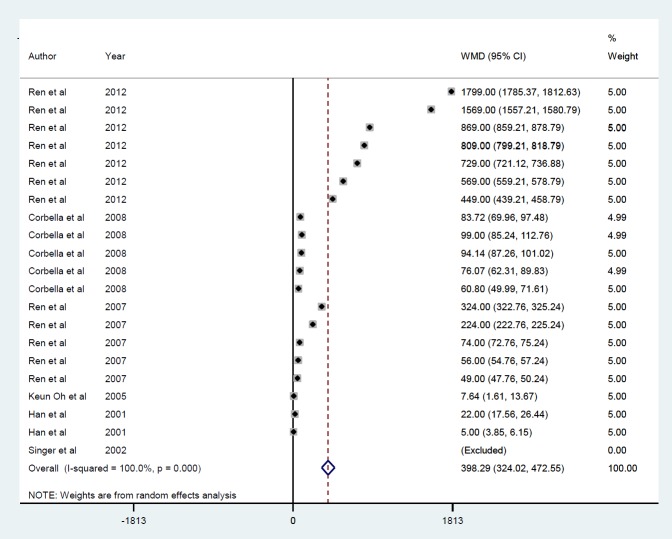
Mean of insulin level after insulin gene therapy by viral vectors

**Figure 10:**
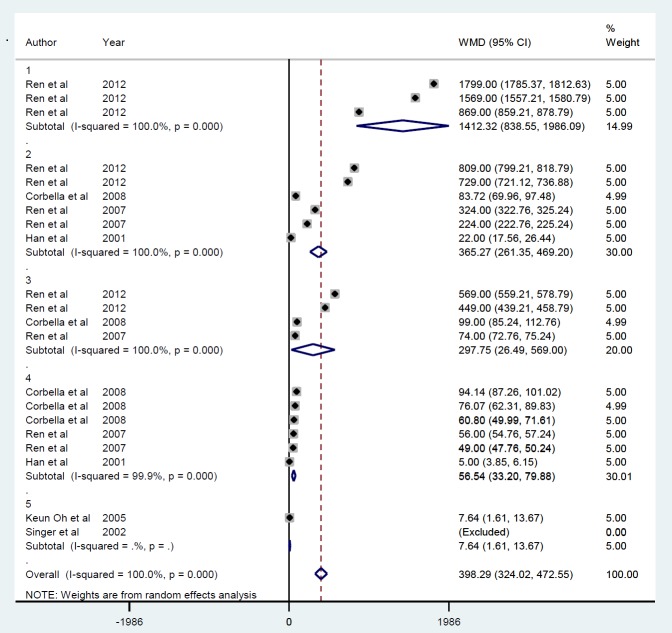
Subgroup analysis by follow-up duration for the mean of insulin level after insulin gene therapy by viral vectors

Meta-analysis of 6 studies (21, 31, 32, 34-36) with 20 datasets describing the effects of insulin gene therapy with lentiviral carriers on blood insulin showed a mean increase of 398.3 pmol/l (*P*<0.001) (Figure 9). There was no publication bias (*P*=0.06) and overall heterogeneity (I^2^=100%), as well as between-study heterogeneity for the duration of measurement, i.e., ≤ 10 min (ID=1), ≥ 15 and ≤ 30 min (ID=2), ≥ 40 and ≤ 60 min (ID=3), ≥ 90 and ≤ 150 min (ID=4), ≥ 21, and ≤ 90 days (ID=5) (Figure 10).

*Gene therapy by non-viral vector*

**Figure 11:**
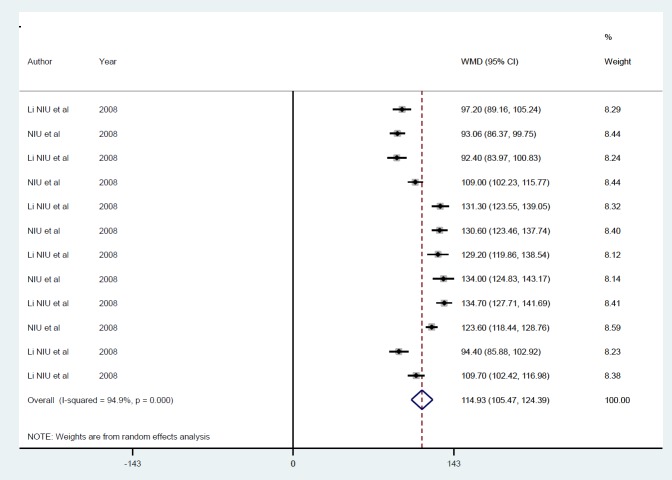
Mean of insulin level after insulin gene therapy by non-viral vectors

**Figure 12:**
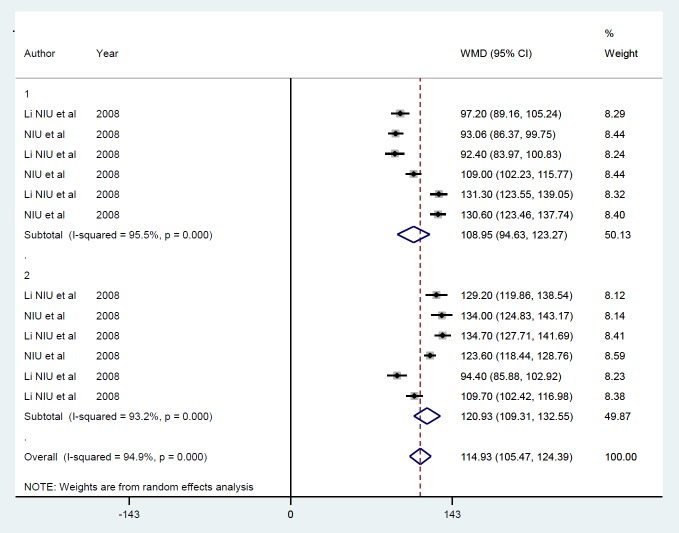
Subgroup analysis by follow-up duration for the mean of insulin level after insulin gene therapy by non-viral vectors

Random-effects meta-analysis confirmed the effects of insulin gene therapy with non-viral carriers on raised blood insulin by 114.9 pmol/l (*P*<0.001) (Figure 11). There was no evidence of publication bias (*P*=0.70), and heterogeneity between studies was high (I^2^=94.9%), which was unaffected by one study or follow-up duration (Figure 12). 

***Findings from the meta-analysis on insulin gene therapy and bodyweight***

*Gene therapy by viral vector*

**Figure 13:**
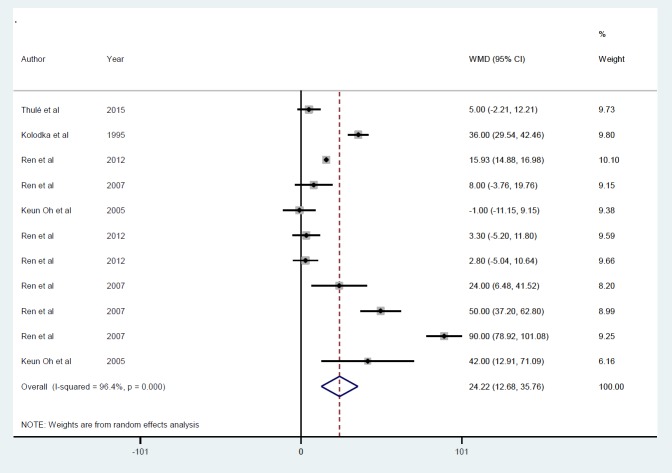
Mean of bodyweight after insulin gene therapy by viral vectors

**Figure 14:**
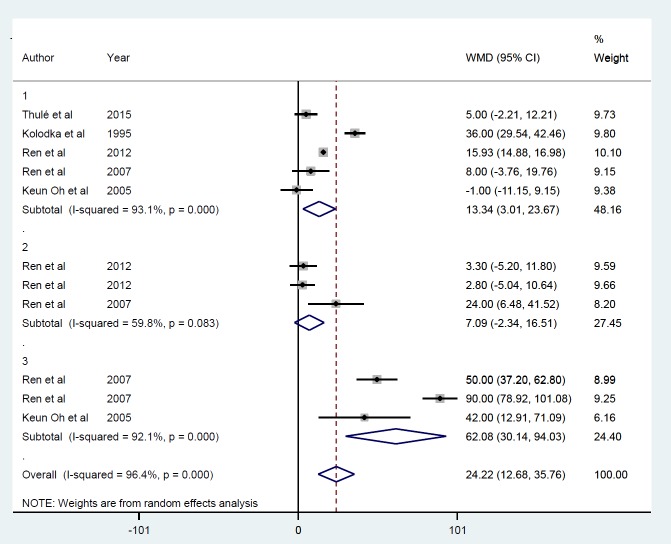
Subgroup analysis by follow-up duration for the mean of weight after insulin gene therapy by viral vectors

Gene therapy by viral vectors increased bodyweight (Figure 13) in 5 studies (21, 24, 25, 29, 31) with 11 datasets, which increased by 24.2 g (*P*<0.001). There was no evidence of publication bias (*P*=0.45), and between-study heterogeneity was apparent (I^2 ^=96.4%, *P*<0.001), with no single study influencing the final effect. Subgroup analysis by follow-up duration showed an increased response with time, i.e., ≤ 5 days (ID=1), 13.3 g, (*P*<0.01); between ≥ 50 and ≤ 70 days (ID=3), 62.1 g, (*P*<0.001); but no effect during ≥ 10 and ≤ 25 days (ID=2) (7.09 g *P*=0.14) (Figure 14). Due to insufficient studies in the AAV and retroviral, subgroup analysis according to gene delivery methods was not performed.

## Discussion

Reducing blood glucose in T1DM is necessary to avoid side effects such as neuropathy, glaucoma, nephropathy, and cardiomyopathy (37-40), for which the most popular treatment is insulin injection, although this is not very practical. It can also cause hyperinsulinemia, which is a risk factor for progressive insulin resistance and cardiovascular damage (41, 42). Therefore insulin gene therapy is currently a focus of future T1DM treatment, with the restoration of a dynamic and more precise method of insulin production (43). The different approaches that can be used to more effectively maintain euglycemia are promoting the survival and proliferation of islets β cells, preventing their destruction by the immune system, and the employment of non-islets β cells such as hepatocytes, myocytes, fibroblasts, and intestinal and gastric epithelial cells to regulate insulin release (20). Also, gene targeting in T1DM can be achieved using viral or non-viral vectors (43, 44), for which the former is more effective (45). We have performed the first systematic review and meta-analysis to investigate the efficiency of insulin gene therapy for IPGTT, FBS, insulin, and bodyweight. 

IPGGT studies on streptozocin (STZ)-induced diabetic mice treated with the insulin gene, delivered intrapancreatically by recombinant Ad (rAD) vector, corrected hyperglycemia and glucose tolerance (31). This response was, however, transient and typically persisted for only 1–3 weeks (20). Studies on STZ-induced diabetic rodents treated with the insulin gene showed that all gene therapies decreased blood glucose and increased insulin. Because lentiviruses and retroviruses are integrative vectors, they can elicit long-term benefits, as shown in rats (29) and mice (21). In both studies, an HMD/INS-FUR construct using INS-FUR was cloned into the site of LV HIV/MSCV (HMD) and injected into the portal vein by intervallic fusion to be delivered into the liver. In STZ-induced diabetic rats, blood glucose was returned to normal for at least 500 days without any adverse response. 

One of the other factors associated with insulin gene therapy for T1DM is increased bodyweight, as diabetes improves, as confirmed by our meta-analysis. We also showed significant effects on FBS and blood insulin by non-viral vectors. Until recently, a major limitation of viral insulin therapy has been the lack of meal-dependency on insulin secretion in these surrogate cells. K-cells are native endocrine cells that are glucose-responsive native endocrine cells, located primarily in the stomach, duodenum, jejunum, and gut hormone GIP (46), which normally potentiates postprandial insulin release (27). It has therefore been proposed that K-cells may be suitable targets for T1DM insulin gene therapy (47), although they have a short lifespan of 3–5 days, which necessitates frequent and repeated gene administration. Taking all these studies together, we observed substantial heterogeneity due to animal type, sample volume, the method of determining T1DM and the gene delivery method. Therefore, the random effect model was used to reduce these contrasting effects but could not find the sources of the heterogeneity, which suggests that the efficacy of insulin gene therapy is variable. In spite of these limitations, including publication bias, our study had several strengths, being the first meta-analysis focused on the effects of insulin gene therapy on T1DM related factors. 

**Table 1:** Gene therapy for type 1 diabetes mellitus using non-viral vectors

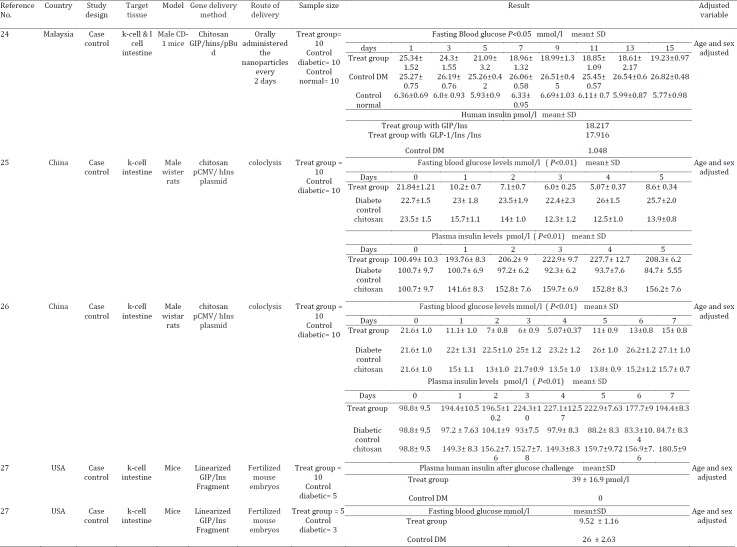

**Table 2:** Gene therapy for type 1 diabetes mellitus using viral vectors

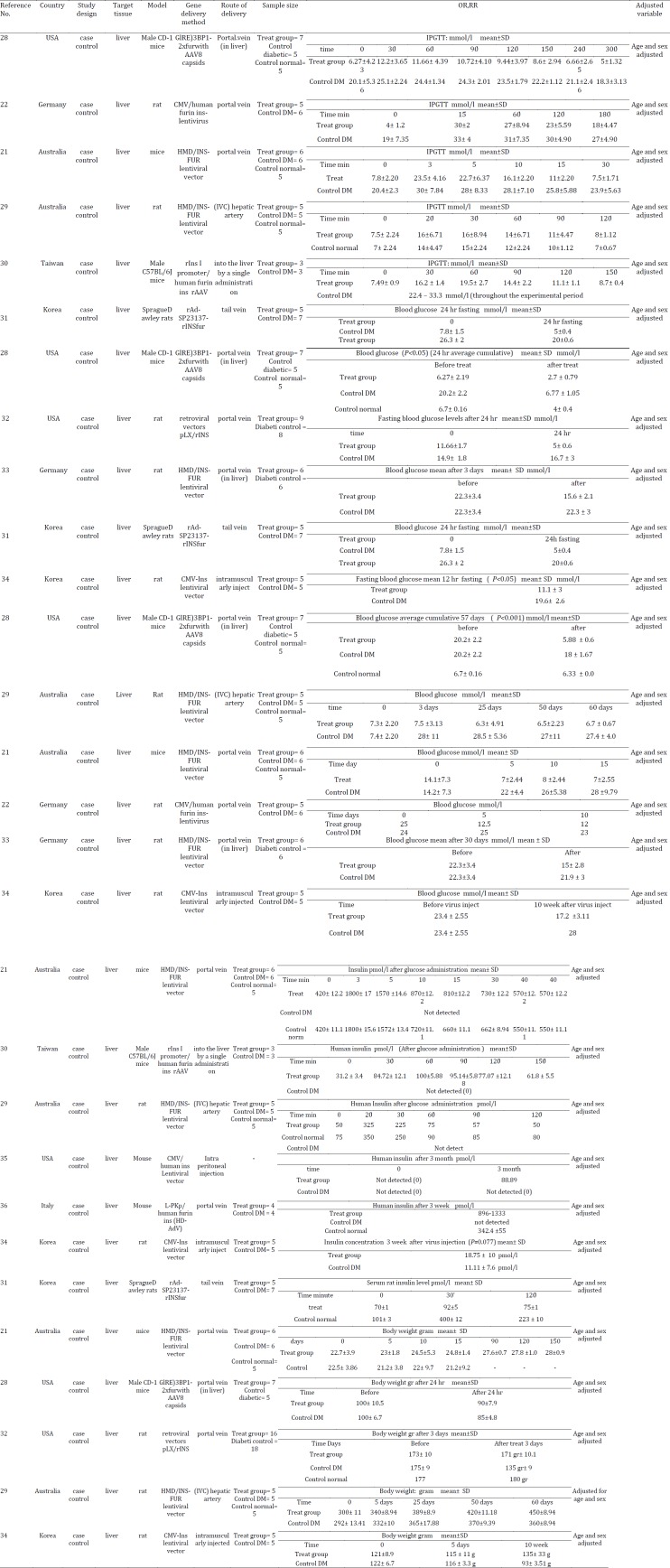

## Conclusion

The meta-analysis findings showed a significant effect for insulin gene therapy and T1DM related factors, including IPGTT, fasting blood glucose, insulin, and bodyweight in diabetic rodents. 
